# Robotic-Assisted Thoracoscopic Circumferential Resection of Congenital Esophageal Stenosis in Under 10 kg Patient

**DOI:** 10.1055/a-2905-8655

**Published:** 2026-07-24

**Authors:** Francesco Fascetti-Leon, Federica Varner, Federica De Corti, Luca Maria Antoniello, Luisa Meneghini, Alvise Guariento, Miriam Duci

**Affiliations:** 1 Division of Pediatric SurgeryDepartment of Women and Children Health 9308 Università degli Studi di PadovaPadovaVenetoItaly; 2 Division of Women's and Children's HealthPediatric Surgery UnitPadova University HospitalPadovaItaly; 3 Anesthesiology Pediatric UnitDepartment of Women's and Children's Health 9308 University of PaduaPadovaVenetoItaly; 4 Division of Cardiovascular Surgery 9308 Università degli Studi di PadovaPadovaItaly

**Keywords:** robotic surgery, esophageal surgery, children

## Abstract

Congenital esophageal stenosis is a rare and heterogenous malformation often associated with esophageal atresia (EA). Failure of conservative endoscopic treatment leads to surgery. Transthoracic approach is rarely advocated due to the peridiaphragmatic localization of the stricture. Robotic-assisted thoracoscopic surgery (RATS) may offer enhanced precision in confined spaces; its application in patients under 10 kg is still considered a challenge. We report the first description of RATS resection of cartilaginous congenital stenosis and esophagoesophagostomy in a 9.5 kg patient. A female patient with a history of EA type III was corrected via videothoracoscopy, two cardiac surgeries via sternotomy, and laparoscopic treatment of duodenal atresia. During weaning, she did not tolerate thickened food, and endoscopy revealed a patent anastomosis but a distal esophageal stricture. A course of pneumatic dilation was attempted. Persistent clinical and radiological findings indicated the need for surgery. At 15 months, the patient underwent three-trocar thoracoscopy with the da Vinci Xi system. Esophagoscopy helped the identification of the stricture. A longitudinal incision exposed a 2-cm segment of thickened esophageal wall requiring complete excision. A tension-free end-to-end anastomosis was performed using 4/0 PDS. Tracheobronchial remnants were confirmed at histopathologIcal finding. Esophageal contrastography demonstrated a good diameter anastomosis. One month after the operation, she was able to swallow solid food. Surgery for congenital stenosis is indicated when endoscopic management fails. RATS for esophageal diseases in patients under 10 kg is feasible, and peridiahragmatic esophagus particularly suits to this technique. Previous accesses to the thorax seems not to be a limitation.

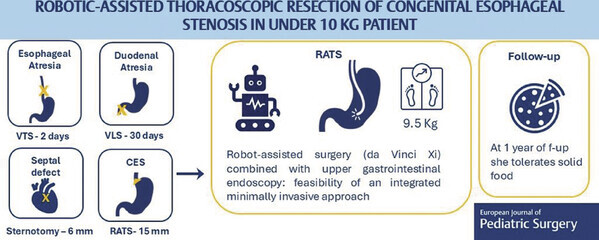

## Introduction

Congenital esophageal stenosis (CES) is a rare and heterogenous malformation often associated with esophageal atresia (EA), whose management is still far from being standardized. 1 2 The current literature on CES treatment is limited and heterogeneous, making it difficult to define an evidence-based diagnostic and therapeutic pathway. 3 Endoscopic dilation is generally considered the first-line approach, particularly in the membranous and fibromuscular subtypes. 4 5 6 However, when conservative treatment fails, cartilaginous nature of CES—due to tracheobronchial remnants—is hypothesized, and surgery may become necessary. 2 5 6 The surgical management of CES poses several challenges, especially when the stenotic segment is located near the diaphragmatic hiatus. In such cases, a transthoracic approach is challenging due to the anatomical constraints and technical difficulties involved. 2 6 Robotic-assisted thoracoscopic surgery (RATS) may offer substantial advantages. Despite the potential benefits, the use of RATS in pediatric patients, especially those weighing less than 10 kg, is still limited. We report the first description of RATS resection of cartilaginous congenital stenosis and esophagoesophagostomy in a 9.5 kg patient.

## Case Report

**Video 1** The video illustrates the key steps of the procedure.


**Fig. 1:**
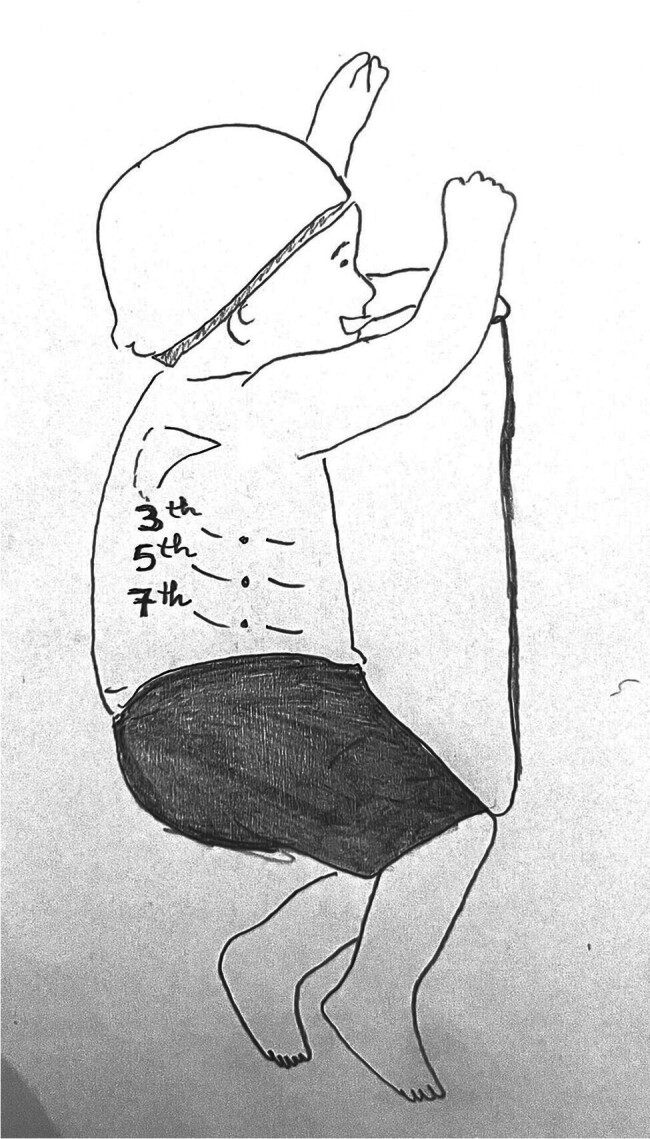
Patient position.

**Fig. 2:**
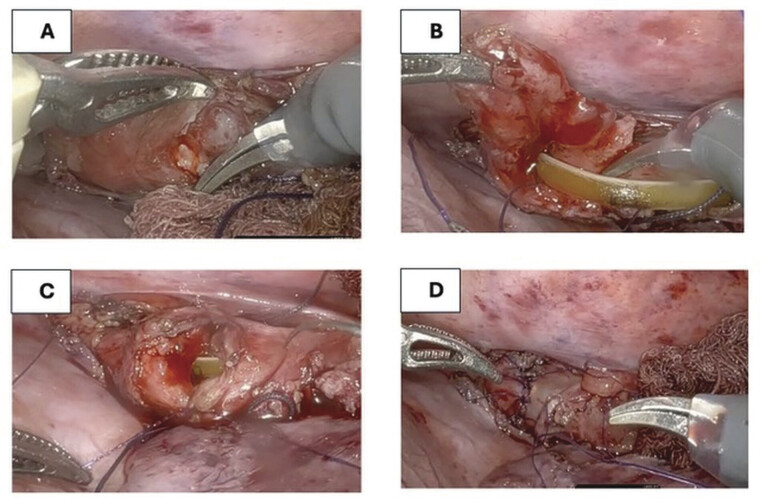
Freeze frames of the surgical procedure. ( **A** ) Longitudinal esophagotomy initiated at the level of the stenosis, guided by the externally visible indentation and intraoperative endoscopic correlation. ( **B** ) Circumferential resection of the stenotic esophageal wall. ( **C** ) Reconstruction phase: tension-free esophagoesophagostomy performed using interrupted sutures. ( **D** ) Completed anastomosis: final appearance after suturing, with endoscopic check.

A female patient with a history of type III EA repaired thoracoscopically on day 2 of life and also underwent two cardiac surgeries in the first 4 months to correct associated congenital heart defects. Following EA repair, she developed persistence feeding intolerance. Diagnostic workout raised suspicion of postpyloric obstruction. Laparoscopy confirmed duodenal stenosis, and a side-to-side duodenoduodenostomy was performed. During the weaning period, the patient poorly tolerated thickened feeds. Endoscopy showed a patent esophageal anastomosis but identified a distal stricture. Three pneumatic dilations were performed (up to 8 Ch, 6 atm), without clinical improvement. Imaging confirmed persistent stenosis, and magnetic resonance imaging (MRI) suggested CES without clear cartilaginous rings. At 15 months, due to failed conservative treatment and suspected CES with tracheobronchial remnants, robotic-assisted thoracoscopic resection was performed using the da Vinci Xi system ( Fig. 1 ). The patient was placed in a left semiprone position chosen to optimize gravitational lung retraction and enhance exposure of the posterior mediastinum in a small thoracic cavity. The procedure was performed under general anesthesia with single-lung ventilation using selective bronchial intubation. Port placement was carefully planned to respect in line preference of Xi ergonomics while minimizing external and internal collisions in the limited intrathoracic space. The first robotic trocar was inserted in the fifth intercostal space along the midaxillary line. Carbon dioxide insufflation was established at 5 mm Hg to facilitate gentle lung collapse and mediastinal visualization. A 5-mm trocar was initially placed in the seventh intercostal space along the same midaxillary line to assess maneuverability and visualize the supradiaphragmatic esophagus; this port was subsequently replaced with an 8-mm robotic trocar. A third robotic trocar was inserted at the third intercostal space along the midaxillary line. The distance between trocars was approximately 3 cm. The robotic cart was positioned from the back of the patients and arms docked on the manual mode. Exposure of the peridiaphragmatic esophagus was facilitated by the articulated instrument moving closely to the diaphragm. Under combined thoracoscopic visualization and simultaneous endoscopic guidance, the stenotic segment was externally identified as a mild indentation of the esophagus wall and the arrest of progression of the endoscope light. Endoscopic transillumination was used to precisely localize the stenosis and guide the longitudinal esophageal incision. In anticipation of potential complications, a predefined strategy was adopted: low-energy bipolar cautery was used for hemostasis to control minor bleeding, whereas readiness for temporary esophageal traction sutures or conversion to an open approach was maintained in the event of uncontrolled bleeding or inadequate esophageal mobilization. Intraluminal inspection revealed a markedly thickened and stenotic esophageal wall extending approximately 2 cm distally. After careful identification and preservation of the anterior and posterior vagus nerves, a stepwise resection of the abnormal esophageal wall was performed. The excised tissue was firm and contained whitish nodular areas, consistent with cartilaginous remnants. Resection proceeded until normal, elastic esophageal tissue was encountered. ( Fig. 2 ) An 8-Ch nasogastric tube was inserted under direct vision, and a tension-free end-to-end anastomosis was performed using interrupted 4–0 PDS sutures. An intraoperative air-leak test demonstrated no evidence of leakage, and endoscopic assessment confirmed satisfactory anastomotic patency. A 10-Ch thoracic drain was placed at the conclusion of the procedure ( Video 1 ). Histology confirmed tracheobronchial remnants. Recovery was uneventful. Postoperative contrast X-ray demonstrated no evidence of anastomotic leak or residual stricture. The patient progressively resumed oral feeding with good tolerance and was discharged 3 weeks postoperatively. At 6 months of follow-up, upper endoscopy demonstrated an adequate esophageal caliber with no need for dilatation. At 1-year follow-up, the patient was tolerating solid foods without dysphagia or feeding difficulties, with normal growth.

## Discussion

CES is a rare and heterogeneous malformation, often associated with EA, and frequently diagnosed during the course of feeding difficulties or failure of weaning. 2 3 7 Among its subtypes, the cartilaginous form is particularly challenging, as it is typically difficult to objectivate preoperatively and resistant to endoscopic dilation. 2 5 In the absence of standardized treatment protocols, management remains challenging and typically requires a stepwise approach, with surgery being indicated in cases refractory to conservative management. 2 8 In our case, the diagnosis of CES was suspected based on persistent symptoms and combined radiological and endoscopic findings, although MRI was not conclusive in identifying cartilaginous rings. The lack of clinical response to repeated pneumatic dilations, together with the high suspicion of a cartilaginous component, supported the indication for surgical resection rather than further endoscopic attempts, which are known to have limited efficacy and may increase the risk of perforation or progressive fibrosis in this setting. This case was further complicated by the patient's complex surgical history, including prior thoracoscopic EA repair and multiple cardiac surgeries via sternotomy, raising concerns regarding the feasibility of additional minimally invasive procedures in a potentially scarred operative field. The use of RATS in pediatric patients, particularly those under 10 kg, remains relatively uncommon due to technical challenges related to size, instrumentation, working space, and familiarity of pediatric surgeons with robotic approach. 9 However, this technique offers distinct advantages in confined anatomical regions such as the peridiaphragmatic esophagus, where three-dimensional visualization, tremor filtration, and articulated instruments allow precise dissection and reconstruction while preserving critical structures, including the vagus nerves. At the same time, the limitations of RATS must be acknowledged. These include the absence of haptic feedback and the need for meticulous port placement to avoid collisions in small patients. For these reasons, robotic approach in small patients should be considered in complex or reoperative anatomy, and lesions located in confined regions where the technical advantages of robotics may outweigh its limitations. 10 The safe adoption of robotic thoracic procedures in small infants also relies on structured training pathways and institutional experience. A high-volume pediatric minimally invasive surgery program dedicated to robotic training and simulation, progressive case selection, and multidisciplinary collaboration is a fundamental prerequisite. In our center, consolidated expertise in congenital esophageal malformations combined with an established pediatric robotic program was pivotal in extending this approach safely to low-weight patients. Importantly, in this case, previous thoracic access did not pose a limitation, demonstrating that RATS is a viable option even in previously operated patients. Definitive surgical management also enabled histopathological confirmation of the underlying etiology. To our knowledge, this is the first reported case of RATS resection of a cartilaginous CES with esophagoesophagostomy in a pediatric patient under 10 kg. This experience suggests that, in selected cases, robotic surgery represents a safe and effective option for complex esophageal procedures in infants, even in the context of multiple prior thoracic interventions. Future studies and accumulation of clinical experience are necessary to establish clearer guidelines for the surgical management of CES and to define the role of robotic techniques in this setting.

## Notes

**Importance for a Pediatric Surgeon**

- Congenital esophageal stenosis due to tracheobronchial remnants should be suspected in patients with esophageal atresia who present with persistent dysphagia despite repeated endoscopic dilations.
- Robotic-assisted thoracoscopic surgery (RATS) is a feasible and safe minimally invasive approach, even in infants weighing less than 10 kg, particularly for technically demanding distal esophageal lesions and in previously operated patients when performed in experienced centers.
